# Be Considerate; Sanitary Police Work Will Only Grow as We Thoughtfully Direct It

**Published:** 1897-03

**Authors:** 


### BE CONSIDERATE –SANITARY POLICE WORK WILL ONLY GROW AS WE THOUGHTFULLY DIRECT IT.

VETERINARY police and sanitation have been of exceedingly slow growth in this country In most of the countries of Europe there is a thoroughly organized corps of veterinarians engaged, in a civil capacity, in the suppression and extermination of communicable diseases of animals and the protection of the public health from diseases originating in animals. Their system is old; it is thoroughly established; its utility is generally recognized; it is, in most cases, liberally supported, and there is no question as to its general economic and sanitary importance. In most places its scope is being steadily enlarged. In the United States, on the other hand, work of this character has met with a multitude of embarrassments and obstacles, nearly all of which can be traced to the lack of appreciation of it upon the part of the public, which, in turn, is due to lack of information regarding it. The education of the public, or the arousing of public sentiment, is the first step toward all American reforms; but the education of the public is a slow process. It must be done carefully, conservatively, systematically, and when once accomplished the movement that has gained strength and position by virtue of it is established on such a firm foundation that it cannot be shaken. A small beginning in the work of veterinary sanitation and police has been made in some American States and cities. If this work is carried on carefully, economically, and successfully, other work of a similar character is sure to follow; it will increase as knowledge of it grows, until finally there will be a far-reaching system that will lead to the gradual suppression of many of the infectious diseases that afflict animals, the protection of the public health to a very great extent, the saving of innumerable dollars and many lives; all of which will make new openings for veterinarians and lead to a higher development of our profession than it has yet attained.

On the other hand, if the work that is now going on is done in a careless, bungling way; if, instead of public support, active antagonism is aroused; if the work is expensive and it is not made clear that the gain is in proportion to the cost; and, *above all*, if it appears that the work is done by the veterinarian for himself rather than for the public, our progress will end at this point. The veterinary profession will receive a blow that it cannot recover from for many years, and not only will the existing generation of veterinarians suffer, but the growth and importance of the veterinary schools will lag and diminish, and the public, the live-stock interests, the various commonwealths, and the nation will lose through not having this important work carried on in a proper manner.

The occasion of these thoughts is the apparent inclination on the part of some veterinarians in some of our States to regard veterinary sanitary work as a "good thing" that must be made to yield a maximum amount to them in direct monetary return; and those who have the management of this work are greatly harassed and hampered by the extravagant bills and the importunities for more money from some of those whom it is necessary to employ.

In most of the States where veterinarians are employed in public service the rate of compensation is fixed at five dollars per day; and when one considers the importance of doing this work in an economical manner, the fact that not only its development but its continuation, and in many cases its very existence, depend upon this point, and when one considers the secondary advantages that accrue to the veterinarian from doing it, beyond and above the mere money received for his labors, the allowance seems to be sufficiently generous for work of a routine character that is incidental to a general practice, and we can at this time and under these conditions have no sympathy with the clamor for a larger allowance.

Gentlemen, do all that you can to carry this work along economically, conservatively, and successfully. Do not regard the dollar so attentively that all consideration for the future is lost. This work must grow; the country needs it; hasten its growth, generously and willingly.

